# The effect of different levels of dietary restriction on glucose homeostasis and metabolic memory

**DOI:** 10.1007/s11357-018-0011-5

**Published:** 2018-02-17

**Authors:** Stephanie Matyi, Jordan Jackson, Karla Garrett, Sathyaseelan S. Deepa, Archana Unnikrishnan

**Affiliations:** 1 Department of Geriatric Medicine, University of Oklahoma Health Sciences Center, Oklahoma City, OK USA; 2 Reynolds Oklahoma Center on Aging, Oklahoma City, OK USA; 3 Harold Hamm Diabetic Center, Oklahoma City, OK USA

**Keywords:** Dietary restriction, Gene expression, Glucose tolerance, Metabolic memory, Adiposity

## Abstract

Over the past 50 years, dietary restriction (DR) has been shown to extend the life span of a wide variety of organisms. A hallmark feature of DR is improved glucose homeostasis resulting in increased glucose tolerance and insulin sensitivity of animals ranging from rodents to humans. In this study, we demonstrate the early effects of varying levels of DR on glucose tolerance. Within 10 days of 40% DR, glucose tolerance was significantly improved and by 120 days; 10 and 20% DR also showed enhanced glucose tolerance. All three levels of DR showed reduced adiposity, increased expression of genes involved in fat turnover, and a reduction in the expression for markers of inflammation. Studies have shown that mice fed a DR diet retained metabolic memory in terms of improved glucose tolerance even after DR is discontinued. We show that 40% DR not only has an early effect on glucose tolerance but also maintained it after DR was discontinued for 2 months. Therefore, improvement in glucose tolerance is brought about by all three levels of DR but the metabolic memory is not dose responsive.

## Introduction

The first and most studied manipulation shown to increase life span in mammals is dietary/caloric restriction. Dietary restriction (DR) was first shown to increase the life span of rats and subsequently various strains of mice. Research over the past two decades shows that DR increases the life span of a wide variety of other organisms ranging from invertebrates, such as yeast, *C. elegans*, and *Drosophila*, as well as spiders and rotifers to various strains of rats and mice (Weindruch and Walford 1988; Swindell 2012). DR has also been reported to increase the life span of other types of mammals such as Labrador Retrievers (Kealy et al. 2002) and Rhesus monkeys (Colman et al. 2009).

The standard DR diet that is used in most studies with rats and mice is 40% DR, where rodents are fed 60% of the diet consumed by animals fed ad libitum (AL). It is generally believed that increasing the level of restriction leads to a greater increase in life span up to a point (around 60% DR) and where further restriction is harmful (Weindruch et al. 1986; Clancy et al. 2001). For example, Weindruch et al. (1986) reported that a significant increase (over 20%) in the mean survival occurred between ~25 and ~55% DR for female C3B10RF1 mice. However, two recent studies suggest that lower levels of DR are as effective in increasing life span as 40% DR. Our group showed that 10% DR significantly increased the life span of F344 rats to a level that was similar to the increase in life span observed with 40% DR (Richardson et al. 2016). Additionally, Mitchell et al. (2016) showed that 20% DR was as effective and in some cases more effective than 40% DR, at increasing life span in C57BL/6 and DBA/2 mice.

Although recent evidence suggests that levels of DR less than 40% may be just as effective in increasing life span as 40% DR, there is very limited information on the effect of various levels of DR on parameters that might be involved in the life-extending action of DR. The purpose of this study was to investigate the effect of various levels of DR on glucoregulation because one consistent observation in mammals is that DR has a dramatic effect on insulin sensitivity, and it has been argued that improved insulin sensitivity plays a role in DR’s life-extending action (Barzilai et al. 1998). In 1992, Masoro et al. showed that 40% DR significantly reduced and maintained the levels of plasma glucose and insulin at low levels throughout the life span of male F344 rats. McCarter et al. (2007) showed that 40% DR significantly reduces plasma glucose and insulin levels in the male C57BL/6 mice. Furthermore, studies have also shown that both short-term and long-term 40% DR significantly improves glucose tolerance and insulin sensitivity in laboratory rodent models (Escriva et al. 2007; Cameron et al. 2012; Selman and Hempenstall 2012; Mitchell et al. 2016). DR has been shown to have a similar effect in non-human primates. In a 8.5-year follow-up study on aging Rhesus monkeys, Gresl et al. (2001) showed that DR increased insulin sensitivity, increased plasma glucose disappearance rate, and reduced fasting plasma insulin and insulin response to glucose, protecting against the development of insulin resistance. Studies from humans have also shown that DR reduces the levels of blood glucose and fasting plasma insulin/c-peptide and improves insulin sensitivity (Xu et al. 2015; Larson-Meyer et al. 2006).

The purpose of the experiments described below were to determine how quickly various levels of DR (10, 20, and 40%) improve glucose tolerance. We observed that within 10 days of 40% DR, glucose tolerance is improved significantly, and by 4 months, mice fed 10 and 20% DR had a similar improvement in glucose tolerance as 40% DR. We also observed that short-term 40% DR (4 months) imparts a metabolic memory to the mice when they were switched to AL feeding, which was not observed in mice fed 10 and 20% DR.

## Methods

### Animals and diet

Male C57BL/6 mice were purchased from the Jackson Laboratory (Bar Harbor, ME) and housed in the animal facility at the University of Oklahoma Health Sciences Center and maintained under SPF conditions in a HEPA barrier environment. The animals were maintained under temperature- and light-controlled conditions (12:12-h light-dark cycle). The animals were fed irradiated NIH-31 mouse/rat diet from Teklad (Envigo, Madison, WI) until 4 months of age. At four months of age, the mice were separated into four dietary regimens: ad libitum (AL; *n* = 15), 10% DR (*n* = 15), 20% DR (*n* = 15), and 40% DR (*n* = 15), where the DR groups were fed 90, 80, or 60%, respectively, of the food consumed by the AL animals. Food consumption of the AL mice was determined every other week. DR was conducted as previously described (Ikeno et al. 2005; McCarter et al. 2007) housing five mice per cage. The DR mice were fed at 6:00 pm just before the start of the light cycle, which is from 6:00 pm to 6:00 am. After 4 months of DR, five animals from each group were fasted overnight, sacrificed and tissues harvested (epididymal and subcutaneous white adipose tissue), snap frozen in liquid nitrogen, and stored at − 80 °C until used. The AL animals were also fasted overnight for about 14 h along with the DR mice to bring all of them to the same metabolic state. The remaining animals from each group (*n* = 10) were used for longitudinal glucose tolerance tests and body composition. After 4 months of DR, the DR groups were switched to AL feeding for 2 months. All procedures with mice were approved by the Institutional Animal Care and Use Committee at the University of Oklahoma Health Sciences Center.

### Body composition

Body composition of the animals was measured using nuclear magnetic resonance spectroscopy (NMR-Bruker minispec) following DR and 2 months after switching to AL feeding. Body fat and lean body mass of the animals in each group were measured.

### Real-time PCR

The levels of specific messenger RNA (mRNA) transcripts of genes involved in inflammation, fatty acid metabolism, and adipocyte differentiation were measured by real-time PCR in the epididymal and subcutaneous white adipose tissues from DR and AL mice 4 months after the initiation of DR (*n* = 5 per group). Briefly, RNA was isolated using the RNeasy Kit from Qiagen (Germantown, MD, USA). The first-strand cDNA was synthesized from 1 μg RNA using random primers (Promega, Madison, WI, USA) and purified using the QIAquick PCR Purification Kit (Qiagen, Germantown, MD, USA). Expression of the candidate genes were quantified using real-time PCR with SYBR Green, and the primer sequences are given in Table 1. The gene transcripts were normalized to β-actin. Relative gene expression was quantified as comparative Ct analysis using the 2^−ΔΔct^ analysis method with β-actin as endogenous control. One-way ANOVA design with Tukey’s multiple test correction was used to statistically analyze individual samples.

**Table 1:** Primer sequences

Gene name	Forward primer	Reverse primer
Adiponectin	5′-GCCGCTTATGTGTATCGCTCAG-3′	5′-GCCAGTGCTGCCGTCATAATG-3′
Leptin	5′-TGACACCAAAACCCTCATCA-3′	5′-TCATTGGCTATCTGCAGCAC-3′
IL-6	5′-TGGTACTCCAGAAGACCAGAGG-3′	5′-AACGATGATGCACTTGCAGA-3′
TNF-α	5′-CACAGAAAGCATGATCCGCGACGT-3′	5′-CGGCAGAGAGGAGGTTGACTTTCT-3′
MCP-1	5′-CCACTCACCTGCTGCTACTCAT-3′	5′-GGTGATCCTCTTGTAGCTCTCC-3′
FAS	5′-GGAGGTGGTGATAGCCGGTAT-3′	5′-TGGGTAATCCATAGAGCCCAG-3′
ACC	5′-GATGAACCATCTCCGTTGGC-3′	5′-GACCCAATTATGAATCGGGAGTG-3′
CPT-1	5′-AAGGGTAGAGTGGGCAGAGG-3′	5′-GCAGGAGATAAGGGTGAAAGA-3′
MCAD	5′-CTAACCCAGATCCTAAAGTACCCG-3′	5′-GGTGTCGGCTTCCAAATGA-3′
LCAD	5′-CTTGCTTGGCATCAACATCGCAGA-3′	5′-ATTGTAGTACGCTTGCTCTTCCCA-3′
PGC-1α	5′-CCCTGCCATTGTTAAGACC-3′	5′-TGCTGCTGTTCCTGTTTTC-3′
PPARγ-2	5′-CGAGGACATCCAAGACAAC-3′	5′-GTGCTCTGTGACGATCTG-3′
CEBP-α	5′-CAAGAACAGCAACGAGTACCG-3′	5′-GTCACTGGTCAACTCCAGCAC-3′
AP-2	5′-TAACCCTAGATGGCGGGGCCC-3′	5′-AACACATTCCACCACCAGCTTGTC-3′
β-Actin	5′-GATGACCCAGATCATGTTTGAGACC-3′	5′-AGATGGGCACAGTGTGGGTGA-3′

### Glucose tolerance test

Glucose tolerance was determined after an overnight fast of mice after 3, 10, 21, and 120 days of DR. Glucose tolerance was also determined on the 40% restricted C57BL/6 mice switched over to AL feeding (DR-AL) with an *n* of 10 per group. Mice were weighed and injected intraperitoneal with 20% glucose (2 g/kg), and blood glucose levels, collected from tail, were measured over a 120-min period using a glucometer (Contour NEXT EZ, Bayer, Whippany, Germany). The area under curve (AUC) for each curve was determined and represented as AUC glucose (mmol × 120 min).

## Results

### Effect of different levels of DR on body composition

**Fig. 1:**
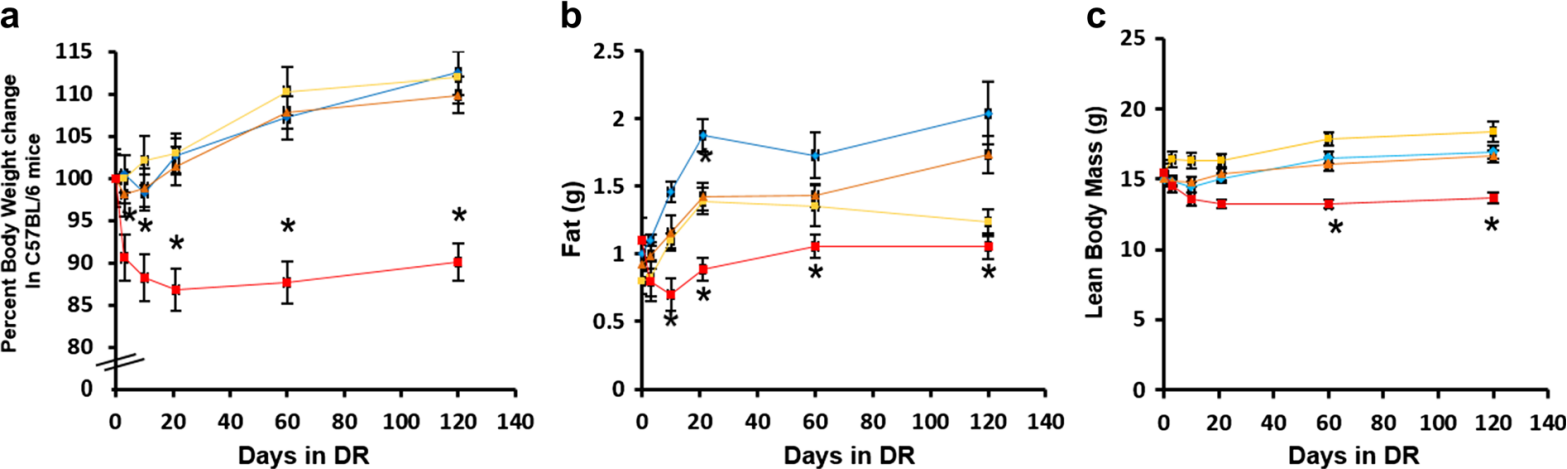
Effect of different levels of DR on body composition. The body weight (**a**), fat mass (**b**), and lean body mass (**c**) of mice on AL and different levels of DR (10, 20, 40%) were measured after 3, 10, 21, 60, and 120 days of DR. Color codes: blue—AL, yellow—10% DR, orange—20% DR, and red—40% DR. Data represented are the mean ± SEM from 10 mice per group and were statistically analyzed by one-way ANOVA with Tukey’s multiple correction test. The asterisk indicates the values that are significantly different (*P* < 0.05) from the AL mice

Studies have previously shown that 40% DR reduces the body weight and total fat mass of laboratory rodents (Barzilai et al. 1998; Mitchell et al. 2016). In this study, we investigated the early effect of DR on the body weight and body composition of male C57BL/6 mice fed three levels of DR (10, 20, and 40% DR). We initiated DR at 4 months of age and followed the changes in body weight and composition over 4 months until they were 8 months of age. Figure 1 shows the body weight and body composition of the animals at 3, 10, 21, 60, and 120 days of DR. Mice fed 10 and 20% DR did not show significant changes in their body weight compared to the AL group at any time point studied (Fig. 1a). On the other hand, mice in the 40% DR group showed a significant decrease in their body weight compared to their AL counterparts starting as early as 3 days of DR. The 40% DR mice exhibited a 10–15% decrease in body weight, which was maintained throughout the 4-month study (Fig. 1a). The total fat mass decreased significantly (~28%) in mice fed 10 and 20% DR after 21 days of DR compared to their AL counterparts (Fig. 1b). Mice fed 40% DR demonstrated a significantly greater reduction in total fat mass (~67%) starting at 10 days of DR (Fig. 1b). The lean body mass of the mice fed 10 and 20% DR did not show any significant change compared to the AL group; however, the mice fed 40% DR had a significant decrease (~20%) in their lean body mass starting at 60 days of DR.

### Effect of different levels of DR on epididymal and subcutaneous fat ratio

**Fig. 2:**
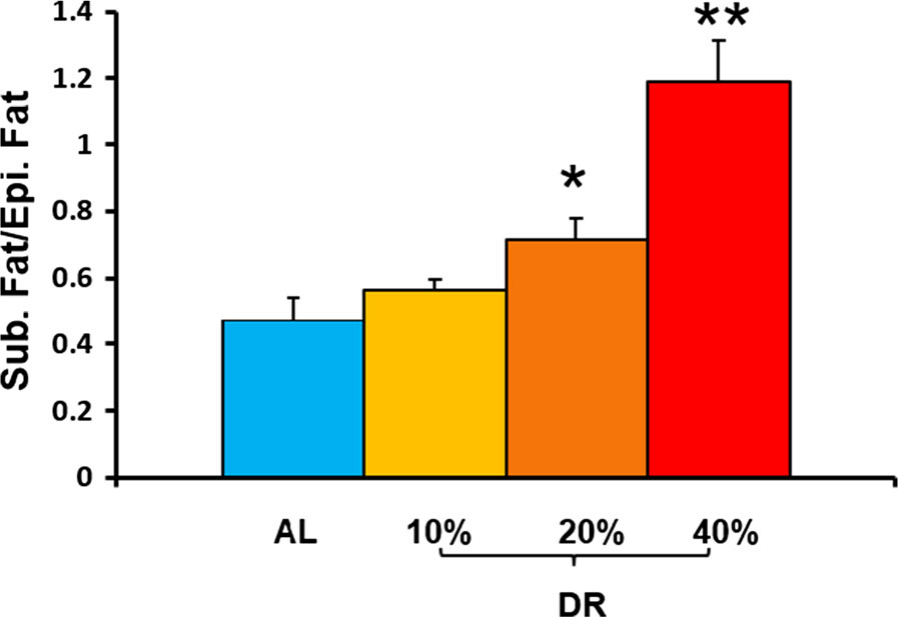
Effect of different levels of DR on ratio of epididymal (Epi) fat to subcutaneous (Sub) fat. The data represented are the mean ± SEM from five mice per group and were statistically analyzed by one-way ANOVA with Tukey’s multiple correction test. The asterisk indicates that the values are significantly different (*P* < 0.05) from the AL mice

Upon showing that the mice on the three levels of DR exhibit a decrease in fat mass as determined by NMR, we were interested in determining the effect of the various levels of DR on specific fat depots because the different fat depots have different phenotypic effects. For example, visceral fat, such as epididymal fat, is associated with metabolic dysfunction and insulin resistance, whereas subcutaneous fat is considered to be protective against the development of insulin resistance (Chau et al. 2014). Additionally, DR has been shown to significantly reduce visceral fat in rats on 40% DR (Barzilai et al. 1998). The data in Fig. 2 shows the ratio of subcutaneous fat to epididymal fat. Mice fed 40% DR exhibited an ~66% increase and mice fed 20% DR showed an ~36% increase in the ratio of subcutaneous/epididymal fat due to a decrease in epididymal fat and no change in subcutaneous fat compared to mice fed AL. In contrast, mice fed 10% DR did not show any significant difference in the ratio of subcutaneous/epididymal fat compared to the AL-fed mice after 4 months of DR. Again, the increase in the ratio of subcutaneous/epididymal fat was due to a reduction of epididymal fat with no significant change in subcutaneous fat. Our data shows that the effect of DR on the ratio of subcutaneous/epididymal fat varies with the level of DR; therefore, the greater the level of DR is, the greater the decrease in epididymal fat is.

### Effect of different levels of DR on the expression of genes in the epididymal and subcutaneous fat depots

**Fig. 3:**
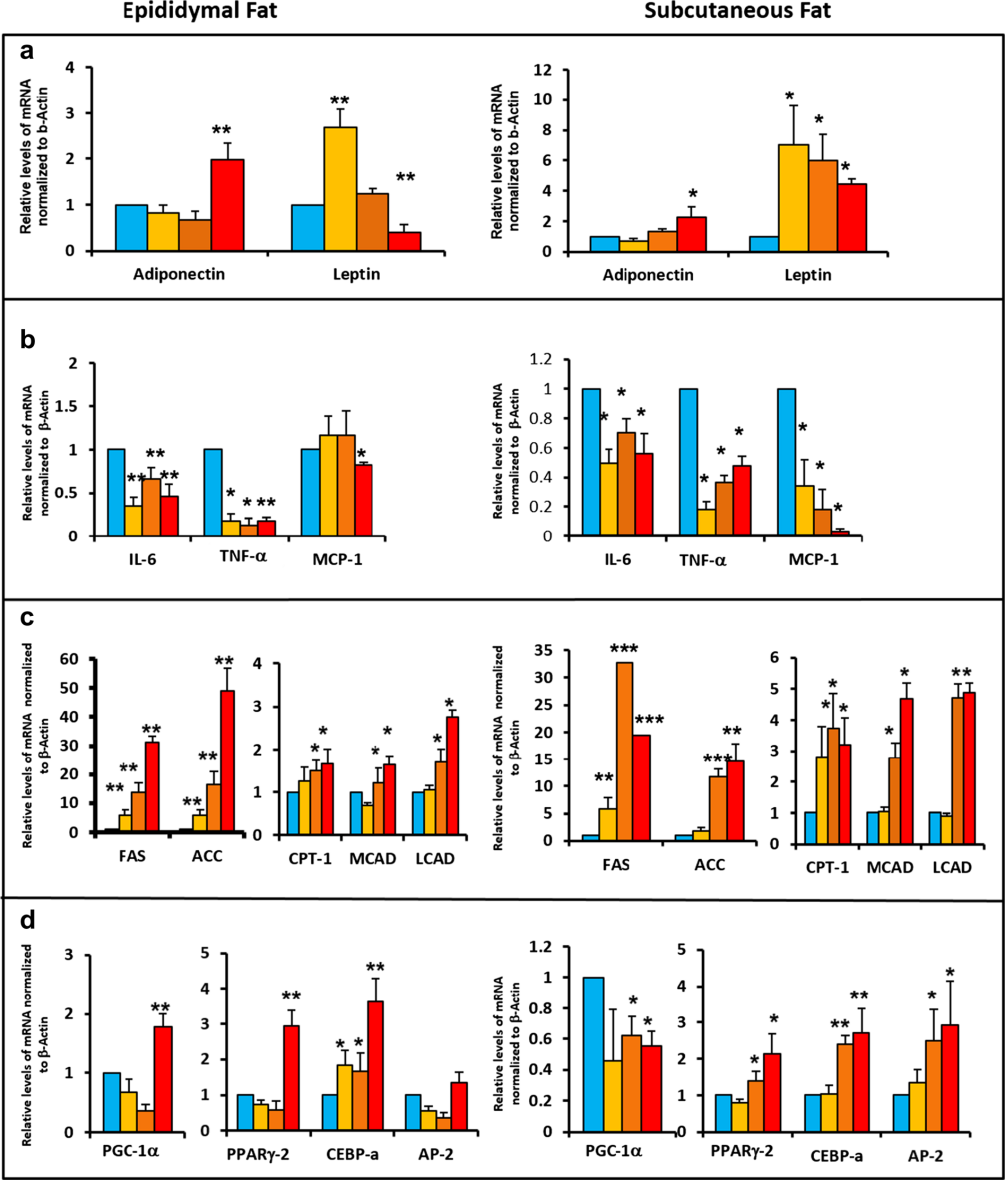
Effect of different levels of DR on gene expression in epididymal and subcutaneous fat. **a** Expression of adiponectin and leptin in both epididymal and subcutaneous fat. **b** Expression of genes involved in inflammation (IL-6, TNF-a, MCP-1) in both epididymal and subcutaneous fat. **c** Expression of genes involved in fatty acid synthesis (FAS and ACC) and fatty acid breakdown (CPT-1, MCAD, LCAD) in epididymal and subcutaneous fat. **d** Expression of genes involved in mitochondrial biogenesis (PGC-1a) and adipocyte differentiation (PPARg-2, CEBP-a, AP-2) in epididymal and subcutaneous fat. Color codes: blue—AL, yellow—10% DR, orange—20% DR, and red—40% DR. The data represented are the mean ± SEM from three to five mice per group and were statistically analyzed by one-way ANOVA with Tukey’s multiple correction test. The asterisk indicates the values that are significantly different (*P* < 0.05) from the AL mice

White adipose tissue is a major endocrine and secretory organ capable of releasing a variety of adipokines such as adiponectin, leptin, IL-6, TNF-α, and MCP-1, all of which are associated with inflammation and inflammatory response (Trayhurn and Wood 2005). These markers of inflammation have been shown to increase with an increase in white adipose tissue mass except for adipokine, which is anti-inflammatory in function (Trayhurn and Wood 2005). As shown in Fig. 3a, 40% DR increased adiponectin expression in both epididymal and subcutaneous fat (100%), which has been reported by Ding et al. (2012). In contrast, 10 and 20% DR did not have any significant effect on adipokine expression. We also measured the gene expression of leptin, the satiety hormone, in both fat depots because DR has been reported to reduce the circulating leptin levels and affect leptin signaling (Shimokawa and Higami 2001). Interestingly, expression of leptin was significantly reduced with 40% DR in the epididymal fat but was significantly increased in the subcutaneous fat (3-fold) (Fig. 3a). Epididymal fat from mice fed 20% DR did not show any significant difference in the levels of leptin mRNA compared to the AL mice, whereas 10% DR increased leptin expression (Fig. 3a). Furthermore, both 10 and 20% DR resulted in a significant increase in leptin expression in the subcutaneous fat (5- to 6-fold) that was much more than the increase in leptin expression observed with 40% DR (Fig. 3a). We next measured the effect of DR on the expression of three pro-inflammatory factors, IL-6, TNF-α, and MCP-1. As shown in Fig. 3b, the expressions of the transcripts of both IL-6 and TNF-α were significantly reduced (≤ 40%) with all three levels of DR in both epididymal and subcutaneous fat. On the other hand, MCP-1 exhibited a differential pattern of expression in the epididymal and subcutaneous fat with only 40% DR showing a significant reduction (25%) in expression of MCP-1 in the epididymal fat and a very dramatic decrease (≤ 70%) in MCP-1 expression with all levels of DR in the subcutaneous fat.

DR has also been shown to regulate the expression of genes involved in fatty acid metabolism (Bruss et al. 2010); therefore, we measured the expression of genes involved in fatty acid synthesis (FAS and ACC) and fatty acid oxidation (CPT-1, MCAD, and LCAD). All levels of DR (10, 20, and 40%) significantly increased the expression of FAS 4- to 30-fold in both epididymal and subcutaneous fat with 40% DR showing the greatest increase in FAS expression in epididymal fat and 20% DR in subcutaneous fat (Fig. 3c). Expression of ACC was also significantly increased 15- to 48-fold in epididymal fat and 10- to 12-fold in subcutaneous fat for mice fed 20 and 40% DR (Fig. 3c). We also measured the expression of three key genes involved in fatty acid oxidation: CPT-1, MCAD, and LCAD. The expressions of these three genes were all increased with DR with each level of DR showing similar response in both epididymal and subcutaneous fat (Fig. 3c). CPT-1 showed an increasing trend in its expression with each levels of DR in both the fat depots, and MCAD and LCAD showed similar expression pattern except with 10% DR, which was not significantly different from its AL counterpart (Fig. 3c).

Short-term 40% DR has been shown to increase markers of mitochondrial biogenesis (e.g., PGC-1α) in many tissues in mice inclusive of white adipose tissue (Nisoli et al. 2005; Nisoli et al. 2003; Larrouy et al. 1999; Higami et al. 2004). In our study, 40% DR significantly increased the expression of PGC-1α by ~75% only in the epididymal fat but not in the subcutaneous fat (Fig. 3d). On the other hand, 10 and 20% DR did not have a significant effect on the expression of PGC-1α in epididymal fat (Fig. 3d). In contrast to the epididymal fat, subcutaneous fat showed a significant decrease (~40–60%) in the expression of PGC-1α for all three levels of DR (Fig. 3d). Finally, we evaluated the expression of genes involved in adipocyte differentiation (PPAR-γ, CEBP-α, and AP-2). Adiponectin has been shown to stimulate adipocyte differentiation (Fu et al. 2005), and in line with this, 40% DR showed a significant increase in the markers of adipocyte differentiation in both epididymal and subcutaneous fat (Fig. 3d), both of which also showed significant increase in adiponectin (Fig. 3a). Epididymal fat from mice fed 10% DR showed a significant increase in CEBP-α but did not show significant difference in the expressions of PPARγ-2 and AP-2, whereas in the subcutaneous fat, 10% DR did not show significant difference in any of the genes studied (Fig. 3d). Similarly, 20% DR also showed only a significant increase in CEBP-α in the epididymal fat, but unlike 10% DR, 20% DR significantly increased PPARγ-2 and CEBP-α in subcutaneous fat (Fig. 3d).

### Effect of different levels of DR on glucose tolerance

**Fig. 4:**
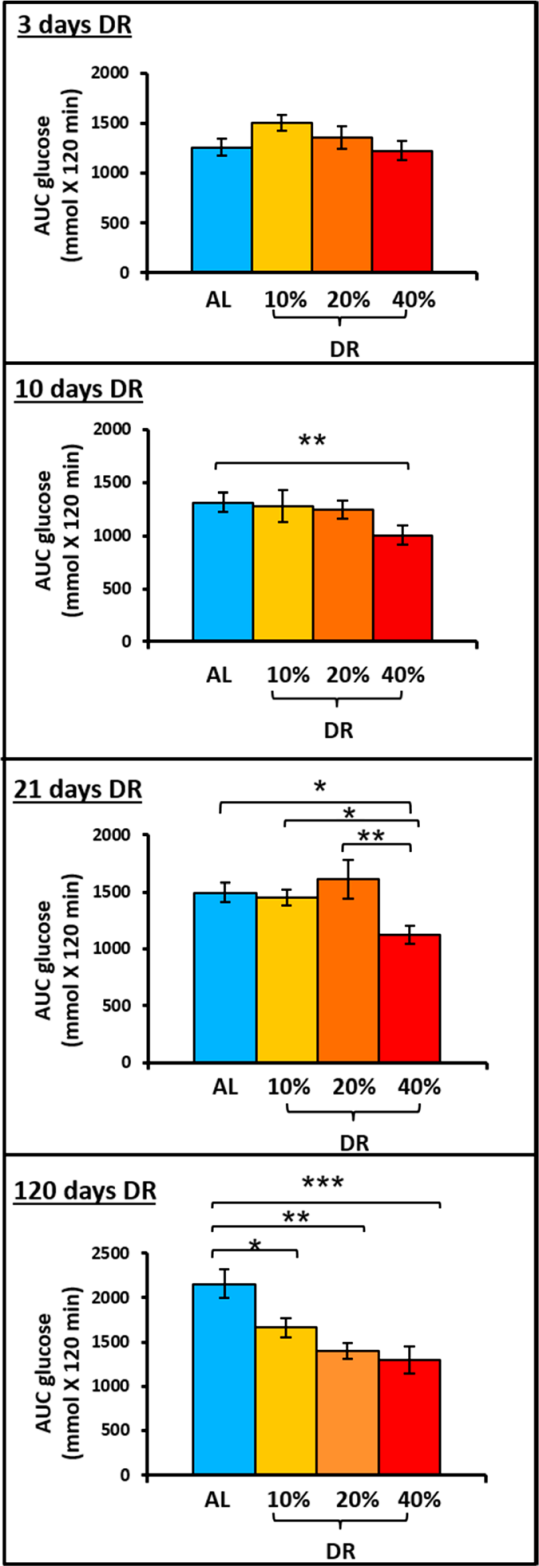
Effect of different levels of DR on glucose tolerance (GTT). The graphs show the area under the curve (AUC) for the GTTs conducted for mice fed DR diets (10, 20, 40%) at 3, 10, 21, and 120 days after the start of DR. Data represented are the mean ± SEM from 10 mice per group except for the 120-day GTT, which had 5 mice per group and were statistically analyzed by one-way ANOVA with Tukey’s multiple correction test. The asterisk indicates the values that are significantly different (*P* < 0.05) from the AL mice for each of the times that glucose tolerance was measured

Our gene expression analyses of both the fat depots show the differential regulation of the different markers of inflammation, fatty acid metabolism, mitochondrial biogenesis, and adipocyte differentiation between the three levels of DR, which could potentially play a role in insulin sensitivity. Data from laboratory rodents and non-human primates have shown that 40% DR consistently reduces blood glucose levels (Masoro et al. 1992; McCarter et al. 2007; Gresl et al. 2001). Further, DR (40%) has been previously shown to improve glucose tolerance in laboratory rodent models (Escriva et al. 2007; Cameron et al. 2012; Selman and Hempenstall 2012; Mitchell et al. 2016); therefore, in this study, we looked at the time course effect of all three levels of DR on glucose tolerance. Figure 4 shows the area under the curves for male C57BL/6 mice fed AL and three levels of DR (10, 20, and 40%). It is evident from Fig. 4 that 40% DR has a dramatic effect on glucose tolerance. Within 10 days of being 40% DR, glucose tolerance was significantly improved (~20%). Mice fed 40% DR for 21 days showed further improvement in glucose tolerance to ~27%, and by 120 days of DR, glucose tolerance was improved ~40% compared to mice fed AL. Thus, 40% DR significantly improves glucose tolerance within 10 days of its implementation, and glucose tolerance steadily improved for the remainder of the study (Fig. 4). As shown in Fig. 4, 10 and 20% DR did not show any significant change in glucose tolerance until 120 days of DR, at which time 10 and 20% DR exhibited improvement (24 and 36%, respectively) in glucose tolerance compared to the AL counterparts. At 120 days of DR, glucose tolerance was not significantly different for the mice fed 10, 20, or 40% DR. Thus, while the kinetics of improving glucose tolerance varies with the level of DR, it appears that a similar level of improvement is achieved within 120 days.

### Effect of different levels of DR on glucose tolerance after switching to AL feeding

**Fig. 5:**
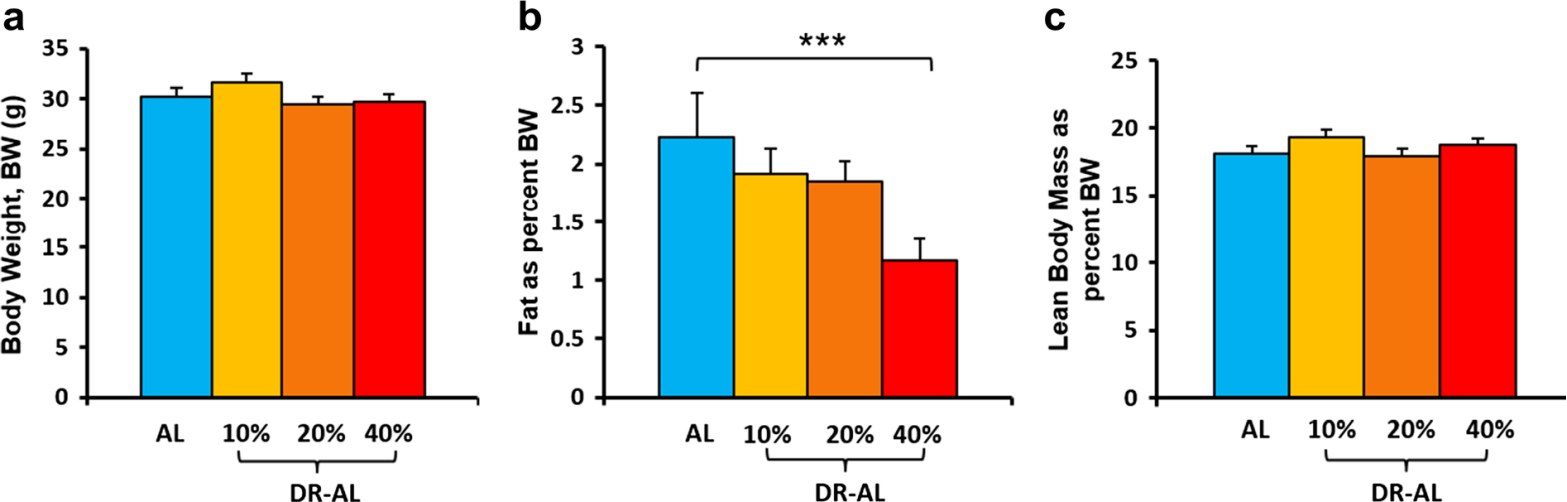
Effect of switching mice fed DR to AL on body composition. Mice fed different levels of DR (10, 20, and 40%) for 120 were fed AL for 2 months. The body weight (**a**), fat mass (**b**), and lean body mass (**c**) are the mean ± SEM from five mice per group and were statistically analyzed by one-way ANOVA with Tukey’s multiple correction test. The asterisk indicates the values that are significantly different (*P* < 0.05) from the AL mice

**Fig. 6:**
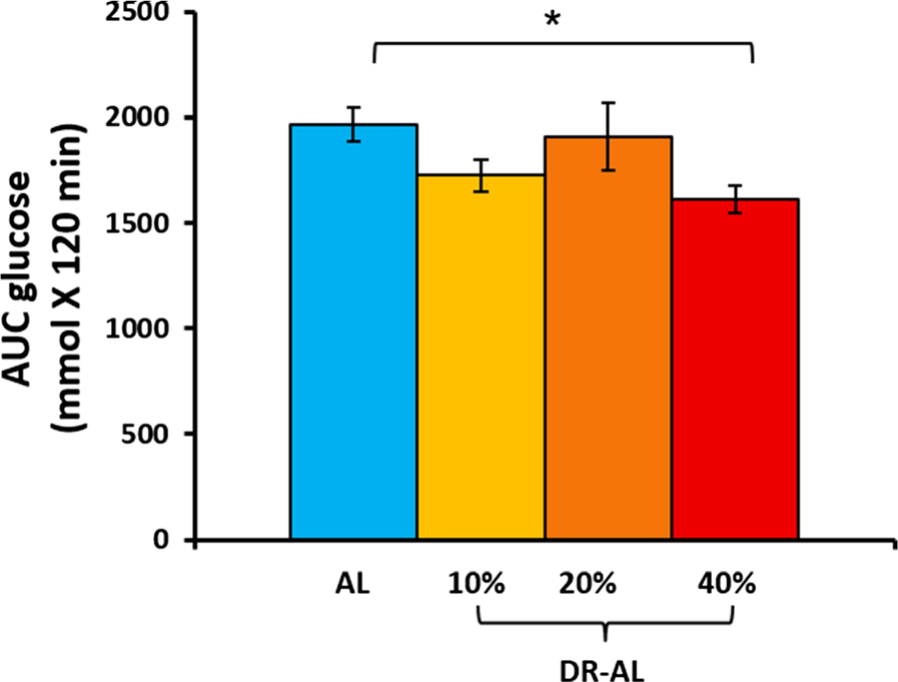
Effect of different levels of DR on glucose tolerance (GTT) after switch to AL diet (DR-AL). GTTs were done after an overnight fast for mice that had been fed the respective DR diets (10, 20, 40%) for 4 months and then fed AL for 2 months. The AUC of the GTTs are shown and are the mean ± SEM from 10 per group and were statistically analyzed by one-way ANOVA with Tukey’s multiple correction test. The asterisk indicates the values that are significantly different (*P* < 0.05) from the AL mice

DR mice have been shown to have metabolic memory (i.e., improved glucose tolerance) after 40% DR is discontinued (Cameron et al. 2012; Selman and Hempenstall et al. 2012). Therefore, we next determined the effect of switching mice fed 10, 20, and 40% DR diet to AL feeding for 5 months (DR-AL) on glucose tolerance. Figure 5 shows the body weight and body composition of the DR animals after switching them to AL feeding. As shown in the Fig. 5a, all three levels of DR demonstrated no significant difference in their body weights compared to their AL counterparts as the 40% DR mice regained their body weight once they were switched to AL feeding. Using NMR to measure body composition, we found that the fat mass of the mice, which had previously been fed 40% DR, remained significantly lower than mice fed AL (Fig. 5b). In contrast, the mice previously fed 10 and 20% DR did not show any significant difference in fat mass compared to mice fed AL, although the level of fat mass trended to be lower than the mice constantly fed AL. Figure 5c shows the lean body mass of the animals switched from a DR diet to AL diet, and the mice previously on the different levels of DR did not show any significant change in lean body mass compared to the AL group. Next, we measured the glucose tolerance in the DR mice after feeding AL for 2 months. The data in Fig. 6 show that the 40% DR group (40% DR-AL) still showed a significant improvement in glucose tolerance (16%) even after DR has been discontinued for 2 months. However, both 10 and 20% DR-AL animals did not show a significant improvement in glucose tolerance after feeding AL for 2 months (Fig. 6). Thus, the mice fed 40% DR for 4 months retained metabolic memory with regard to their fat mass and glucose tolerance, whereas 10 and 20% DR did not.

## Discussion

Dietary restriction without malnutrition is the most robust and reproducible dietary intervention that has been shown to extend life span and delay the onset and progression of most age-related diseases. DR has been shown to increase the life span of a wide variety of animals and is generally considered that the effect of DR on life span is universal. Liao et al. (2010) reported the effect of DR (40% DR) on over 40 different recombinant inbred (RI) lines of male and female mice, and surprisingly, only one third of the mice showed the expected increase in life span on the DR diet; the others either showed no effect or a decrease in life span. It is possible that 40% restriction, which was used by Liao et al. (2010), has a negative effect on the life span of some of the RI lines, but lower levels of DR (10 and 20%) might increase life span for these genotypes. In line with this possibility, two groups have shown that lower levels of DR are as effective at increasing the life span of mice as 40% DR. Richardson et al. (2016) reported that the life span of male F344 rats was not significantly different when fed 10 and 40% DR, and Mitchell et al. (2016) reported that the life span of male and female C57BL/6 mice and DBA/2 mice fed 20% DR was similar or greater than mice fed 40% DR. Therefore, these recent data suggest that lower levels of DR might be as effective at increasing life span as 40% DR. However, there is currently very little information on the effect of low levels of DR (i.e., less than 40% DR) on various processes in rodents.

Many mechanisms have been proposed for the life-extending action of DR; however, one of the hallmarks of DR is improved glucose tolerance and insulin sensitivity, and these changes have been proposed to play a role in the life-extending action of DR (Bartke et al. 2001; Barzilai et al. 1998). Except for the study by Mitchell et al. (2016), all of the research to date on glucose homeostasis and insulin sensitivity has focused on 40% DR. In 2016, Mitchell et al. showed that 20% DR was as effective at improving insulin sensitivity and glucoregulation as 40% DR. For example, blood levels of insulin, glucose, and leptin were reduced (30–99%) after feeding either male or female C57BL/6 mice either 20 or 40% DR for 17–18 months. Insulin resistance, as measured by HOMA-IR, was also lowered in both 20 and 40% DR mice. On the other hand, circulating adiponectin levels were increased with both 20 and 40% DR. We extended the study by Mitchell et al. (2016) by looking at the effect of 10, 20, and 40% DR on glucoregulation and determining how quickly after the implementation of DR that one could observe a significant effect of these levels of DR on glucoregulation. All three levels of DR lead to a significant reduction in total fat mass with the mice with the greatest restriction showing the greatest decrease in fat mass, as would be expected. Interestingly, when we compared the effect of DR on the distribution of fat, we found that the ratio of subcutaneous fat to epididymal fat (i.e., fat that is anti-inflammatory vs pro-inflammatory fat) was significantly increased with 20 and 40% DR, with the ratio higher (~171%) for mice fed 40% DR compared to mice fed 20% DR; 10% DR had no significant effect of the distribution of fat. Thus, the highest level of DR results in a greater reduction in the amount of fat, particularly in the pro-inflammatory epididymal depot.

DR has been shown to reduce adiposity, especially visceral adiposity, partly through the metabolic remodeling of the white adipose tissue (Okita et al. 2015) by increasing the expression of genes involved in fatty acid synthesis in the white adipose tissue and also inducing lipolysis leading to the formation of ketone bodies (Okita et al. 2015; Xu et al. 2015). We observed that all three levels of DR induced the expression of genes involved in fatty acid biosynthesis (e.g., FAS and ACC) with the greatest increase in expression occurring in a dose-responsive manner. However, only 20 and 40% DR significantly increased the expression of the genes (e.g., MCAD and LCAD) involved in fatty acid breakdown.

White adipose tissue previously considered to be an inert tissue mainly involved in energy storage has emerged as a major secretory organ which releases a variety of adipokines that can regulate appetite, energy expenditure, inflammation, glucose homeostasis, and insulin sensitivity (Fantuzzi 2005; Ding et al. 2012). Because DR has been shown to reduce inflammation (Huang et al. 2010) and because fat depot expresses pro-inflammatory cytokines (Trayhurn and Wood 2005), especially in the epididymal fat, we measured the expression of several pro-inflammatory cytokines in epididymal and subcutaneous fat. Interestingly, we found that all three levels of DR reduced the expressions of IL-6 and TNF-α to a similar extent in both epididymal and subcutaneous fat; e.g., the decrease was the same for mice fed 10% DR and 40% DR. On the other hand, expression of MCP-1 was reduced to a much greater extent by 40% DR, especially in subcutaneous fat. Thus, it appears from our limited study of pro-inflammatory factors in fat that a low level of DR is as effective as higher levels of DR in reducing inflammation in fat tissue. It will be of interest in the future to compare the circulating cytokines in mice on various levels of DR over their life span.

It is well established that acute DR improves a range of metabolic parameters in laboratory rodents including improved glucose tolerance (Park et al. 2006; Cameron et al. 2012; Selman and Hempenstall 2012). Interestingly, after 4 months of DR, we observed a similar improvement in glucose tolerance in the three levels of DR. However, our data shows that the time course for the improvement in glucose tolerance differs with respect to the level of DR. Mice fed 40% DR showed a significant improvement in glucose tolerance in 10 days after implementation of DR, while mice fed 10 and 20% DR did not show significant improvement until 4 months of DR. The improvement in glucose tolerance after 4 months is similar for mice fed 10 to 40% DR, which is in agreement with the study by Mitchell et al. (2016), showing that glucose tolerance was similar in mice fed 20 and 40% DR for 17–18 months. Taken together, all three levels of DR show improved glucose tolerance by 4 months of restriction, potentially due to reduced inflammation and controlled fatty acid turnover as observed from our gene expression analysis.

An important aspect of DR is that it can impart cellular/metabolic memory, which can persist even when DR is discontinued, e.g., improved glucose tolerance after DR is discontinued. Selman and Hempenstall (2012) showed that male C57BL/6 mice fed DR for 8 months retained glycemic memory and showed improved glucose tolerance even after they were switched from DR to AL feeding for 10 months. Similarly, Cameron et al. (2012) showed that male C57BL/6 mice retained metabolic memory of 5 months of DR feeding and maintained enhanced glucose tolerance when switched to AL feeding for 3 months. Furthermore, Sadagurski et al. (2014) showed that the pre-weaning food restricted animals have significantly improved insulin sensitivity with increased insulin and glucose tolerance in both male and female mice. Here, we tested the memory effect of DR on all three levels of DR by discontinuing the restricted feeding after 4 months of DR and feeding them AL for 2 months. Mice fed 40% DR showed a significant improvement in glucose tolerance even after 2 months of AL feeding; however, the improvement in glucose tolerance observed in mice fed 10 and 20% DR was lost after the mice were switched to AL feeding for 2 months.

In summary, our data demonstrates that the different levels of DR can have both similar and differential effects. For example, the effect of DR on glucose tolerance after 4 months of DR was similar in mice fed 10, 20, and 40% DR. However, mice fed 40% DR showed rapid improvement in glucose tolerance (within 10 days) months before mice fed 10 or 20% DR. Additionally, glucose tolerance improvement was maintained for 4 months after switching to AL in the mice fed 40% DR but not in the mice fed 10 or 20% DR. While the reduction in adiposity was correlated to the level of DR, expressions of genes involved in fatty acid turnover and inflammation were altered in a similar fashion across all levels of DR. We are currently further characterizing the effect of 10, 20, and 40% DR on insulin sensitivity by measuring it more accurately using the hyperinsulinemic-euglycemic clamp technique, which is the gold standard method used to assess insulin sensitivity in humans and laboratory rodents. To further characterize the mechanism behind the DR-mediated metabolic memory, we are analyzing the DNA methylation profile of the mice fed DR and then switched to AL feeding.
